# Cluster randomized controlled trial of a peer support program for people with diabetes: study protocol for the Australasian peers for progress study

**DOI:** 10.1186/1471-2458-12-843

**Published:** 2012-10-04

**Authors:** Michaela A Riddell, Carla Renwick, Rory Wolfe, Stephen Colgan, James Dunbar, Virginia Hagger, Pilvikki Absetz, Brian Oldenburg

**Affiliations:** 1 School of Public Health and Preventive Medicine, Monash University, Melbourne, Australia; 2 Deakin Health Economics, Population Health Strategic Research Centre, Deakin University, Burwood, Australia; 3 Greater Green Triangle University Department of Rural Health, Flinders University and Deakin University, Warrnambool, Australia; 4 Diabetes Australia – Vic, Melbourne, Australia; 5 National Institute of Health and Welfare (THL), Helsinki, Finland; 6 Research Fellow, International Public Health Unit, Department of Epidemiology and Preventive Medicine, Monash University, Commercial Road, Melbourne, VIC, 3004, Australia

**Keywords:** Peer support, Diabetes, Self-management, Support group

## Abstract

**Background:**

Well managed diabetes requires active self-management in order to ensure optimal glycaemic control and appropriate use of available clinical services and other supports. Peer supporters can assist people with their daily diabetes self-management activities, provide emotional and social support, assist and encourage clinical care and be available when needed.

**Methods:**

A national database of Australians diagnosed with type 2 diabetes is being used to invite people in pre-determined locations to participate in community-based peer support groups. Peer supporters are self-identified from these communities. All consenting participants receive diabetes self-management education and education manual prior to randomization by community to a peer support intervention or usual care. This multi-faceted intervention comprises four interconnected components for delivering support to the participants. (1) Trained supporters lead 12 monthly group meetings. Participants are assisted to set goals to improve diabetes self-management, discuss with and encourage each other to strengthen linkages with local clinical services (including allied health services) as well as provide social and emotional support. (2) Support through regular supporter-participant or participant-participant contact, between monthly sessions, is also promoted in order to maintain motivation and encourage self-improvement and confidence in diabetes self-management. (3) Participants receive a workbook containing diabetes information, resources and community support services, key diabetes management behaviors and monthly goal setting activity sheets. (4) Finally, a password protected website contains further resources for the participants. Supporters are mentored and assisted throughout the intervention by other supporters and the research team through attendance at a weekly teleconference. Data, including a self-administered lifestyle survey, anthropometric and biomedical measures are collected on all participants at baseline, 6 and 12 months. The primary outcome is change in cardiovascular disease risk using the UKPDS risk equation. Secondary outcomes include biomedical, quality of life, psychosocial functioning, and other lifestyle measures. An economic evaluation will determine whether the program is cost effective.

**Discussion:**

This manuscript presents the protocol for a cluster randomized controlled trial of group-based peer support for people with type 2 diabetes in a community setting. Results from this trial will contribute evidence about the effectiveness of peer support in achieving effective self-management of diabetes.

**Trial registration number:**

Australian New Zealand Clinical Trials Registry (ANZCTR); ACTRN12609000469213

## Background

In most countries around the world, diabetes is now one of the leading chronic diseases; around 4.4% of Australians are already diagnosed with diabetes (types 1 & 2) in 2007–08 and this number is continuing to rise [1,2]. In 2003, diabetes was responsible for 5.5% of the total burden of disease in Australia, with 92% of this burden due to Type 2 diabetes (T2DM). People with diabetes are at significantly greater risk of serious complications such as heart attack, stroke, blindness, renal failure and lower-limb amputation [3]. Such complications more than double the cost of care [4].

Due to the chronic nature of diabetes, much of the disease management and glycaemic control needs to be self-managed. The person with diabetes is required to adopt and maintain lifestyle and behaviour changes related to diet and exercise, glycaemic control, medication and integration of clinical care; and collectively, these steps have been shown to reduce risk of diabetes complications [5]. People with diabetes require additional resources and support to facilitate and achieve these changes; and although social and emotional support can occur through family and friends, most people require additional supports as well [6], e.g., from peers.

Peer support refers to the provision of emotional, appraisal and informational support from people who have experiential knowledge of a condition [7]. This support functions to complement, supplement and extend formal primary care services. It relies on non-hierarchical, reciprocal relationships, which provide a flexible supplement to formal health services for people with a chronic disease [7]. In addition, peer support can also foster understanding and trust of health care staff among groups who otherwise may be alienated from or have poor access to health care.

As identified in a World Health Organization (WHO) Report [8] concerning peer support programs, more research is required to identify the key ‘active’ components of these programs and particular features which may contribute to better outcomes. A recent systematic review of peer-led chronic disease self-management programs concluded that such programs may lead to small, short term improvements in participants’ self-efficacy, self-rated health, cognitive symptom management, and frequency of aerobic exercise [9]. There remains a lack of evidence concerning medium and longer-term clinical and other outcomes and there is no unequivocal evidence concerning the long-term impact on appropriate use of health services.

Peers for Progress, a global initiative of the American Academy of Family Physicians Foundation (AAFPF) [10], was developed from a WHO consultation that was held to identify and accelerate global best practices in peer support for health [11]. This global initiative aims to strengthen evidence of the value of peer support through the funding of 14 “evaluation” grants around the world. The Peers for Progress program initially identified three core functions [12] and subsequently added a fourth [11,13] and these are:

i. *Assistance & consultation in applying a diabetes management plan in daily life* – (e.g. goal setting, problem solving, role play behaviours and trouble shooting)

ii. *Social and emotional support* – (e.g. encouragement, empathy, support & skills to deal with stress, simply “being there”)

iii. *Linkages to and assistance in gaining access to clinical care* – (e.g. promote importance of clinical care, encourage development of communication skills to enhance interaction with clinicians)

iv. *Ongoing support –* (e.g. flexible, proactive, as needed, continuing availability)

In spite of diversity of setting and model of peer support being implemented in each country, each of the current trials is evaluating the same four key functions in order to a) provide a template for global standardization and program coherence and, at the same time, b) encourage flexibility for local tailoring for local implementation [11,14].

As the largest non-governmental organisation responsible for diabetes in Australia, Diabetes Australia has representative organisations in each state and territory of the country. Diabetes Australia–Vic (DA–Vic) has been providing financial and technical assistance to an existing community network (ComNet) of peer support groups for people with diabetes for many years and there are over 60 groups throughout the state of Victoria. These groups provide information, resources and support for people with diabetes, their families and carers. A 2005 evaluation of the ComNet program [15] identified several ways in which this program might be further enhanced to improve access to educational resources, connections with local health services and group sustainability. Compared with professionally-led groups, where the focus may be purely education [6], and the unstructured and less formal untrained DA-Vic ComNet support groups, the Australasian Peers for Progress – Diabetes Project (Aust PfP-DP) aims to implement and evaluate peer led, group based peer support in which people with T2DM within a specified local community meet to share their self-management challenges, explore and develop strategies to overcome these challenges and sustain health behaviour change using the experiences and support of the group. This program emphasizes formalized training of voluntary lay supporters/group facilitators with ongoing support and a clear structure, which identifies active components and processes conducive to improving daily management of diabetes, positive behaviour change and linkage and access to clinical care.

## Methods/Design

### Study aims

The aim of this study is to implement and evaluate the extent to which the Aust PfP-DP improves diabetes control by the provision of:

i. Assistance and consultation in applying diabetes management plan in daily life;

ii. Social and emotional support;

iii. Linkages to and assistance in gaining access to clinical care; and

iv. Ongoing availability of support

The primary outcome is cardiovascular disease (CVD) risk using the UK Prospective Diabetes Study (UKPDS) risk equation [16]. We hypothesize that participants receiving the peer support intervention will achieve a significantly greater reduction in CVD risk. Secondary outcomes include biomedical, quality of life (QoL) and psychosocial functioning, physical activity, medication adherence, nutrition and BMI. We hypothesize these secondary measures will be significantly improved at 12 months following baseline measurement in those receiving peer support intervention, compared with those not receiving the intervention. Furthermore, we will evaluate the cost-effectiveness of the program.

### Study design and setting

**Figure 1:**
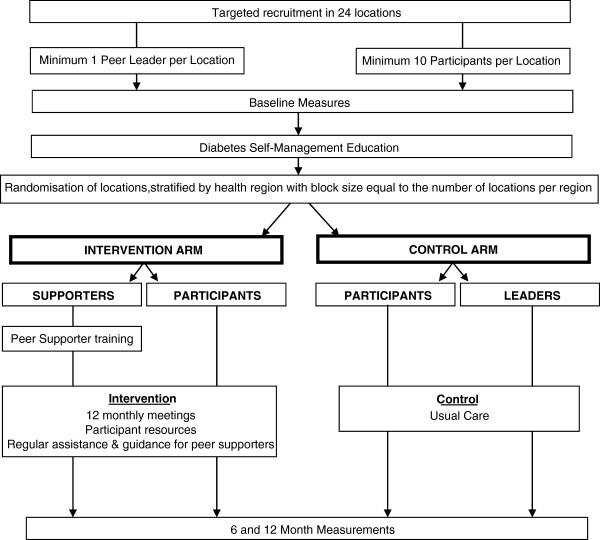
**Australasian Peers for Progress – Consort Diagram for Diabetes Project Study Design**.

The study is a cluster randomized controlled evaluation of a group-based peer support program, implemented and reported in accordance with the requirements of the CONSORT statement [17] and its extension to cluster randomized trials [18]. The study is being conducted in Victoria, Australia, a state that is divided into 79 local government areas (LGAs) within five regional and three metropolitan health regions. Community (defined as a post code area) peer support groups consisting of 10 – 12 participants are recruited, undergo baseline assessment, and then all participants are offered the opportunity to attend a one day diabetes self-management education (DSME) program to ensure that the intervention group does not simply use the sessions to obtain diabetes education rather than peer support. Finally, the groups are randomized to either an intervention arm or usual care arm with group postcode location being the unit of randomization. Groups assigned to the intervention arm participate in the peer support program for 12 months, while those assigned to the control arm continue with their usual care. Figure 1 is a CONSORT diagram of the study design. 

### Ethics approval

The study received ethics approval from the Monash University Human Research Ethics Committee (MUHREC) Project number CF09/1692 – 2009000920. Participants were given a detailed participant information sheet after which signed informed consent from all participants is obtained.

### Eligibility criteria

Participants and peer leaders, able to understand English, aged between 25 – 75 years and diagnosed with T2DM for more than 12 months are eligible. Participants are excluded if they have a current debilitating medical or related condition (e.g. severe mental illness or end-stage cancer).

Self-identified peer supporters are interviewed by the project staff using a set script to assess their current diabetes management regimen, their attitude with respect to the involvement of a clinical care team in diabetes management, their compassion and understanding of difficulties people with diabetes may encounter and their attitude towards referral and seeking assistance when required. Previous life experience, especially working with groups or as community leaders and language abilities is considered favourably.

### Sample size calculations

Sample size was determined by a requirement to have sufficient power to detect a meaningful difference between intervention and control group means of participant UKPDS score changes from baseline to 12 months. During the study, both groups will age by 1 year, which will increase their UKPDS risk calculation by identical amounts [16]. We anticipate this increase would be offset by reductions due to improvements in systolic blood pressure, total cholesterol/HDL cholesterol ratio, HbA_1_C, or smoking status. Assuming a standard deviation of 5% in baseline to 12 month UKPDS changes in both groups, 100 individuals per study arm with 12 month follow-up are required to provide 80% power to detect, with a 2 sided p-value of 0.05 for significance, a mean reduction of cardiovascular risk in the peer support arm that is 2% greater than any mean reduction in the usual care arm. The clustering of individuals by actual peer group (intervention arm) or future group (usual care arm) was not adjusted for because the comparisons across clusters are within-person changes over time and previous studies of UKPDS change in similar clusters found no clustering effects. Hence 12 groups and 120 participants per arm are required at baseline.

### Recruitment

**Figure 2:**
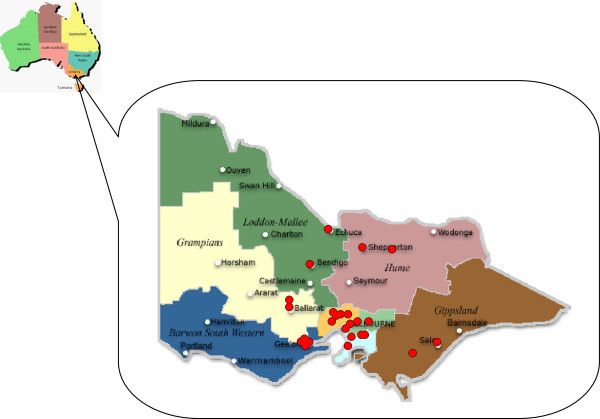
**Australasian Peers for Progress Diabetes Project Group locations in Victorian Health regions.** Two locations were selected for randomisation in four of the five rural health regions. The regional centre of Geelong in the rural health region of Barwon South Western and each of the three metropolitan health regions had sufficient population and area to support the selection and randomisation of four group locations. An additional group was initiated as a pilot group in the western Metropolitan health region.

DA–Vic administers the National Diabetes Services Scheme (NDSS), an initiative of the Australian Government, which contains contact details for more than 85% of all clinically confirmed cases of diabetes (including type 1, type 2 and gestational) in Victoria. Benefits of NDSS registration include access to diabetes related products at subsidized prices [19]. DA–Vic facilitated contact with people diagnosed with T2DM in Victoria and registered on the NDSS database. Diabetes prevalence data from each of the 79 local government areas (LGAs) in Victoria [20] identified 10 regional towns, two from each non-metropolitan Melbourne health region, and 12 metropolitan post codes in the three metropolitan health regions for recruitment. Locations are suitable if the population was more than 10,000 and the density of NDSS registrants was more than 2.5%. Figure 2 shows group location by health region. 

Prospective participants, enrolled on the NDSS database for more than 1 year, who indicated willingness to be contacted for research purposes, are resident within the selected location, and are aged between 25 – 75 years are invited by mail to contact the project team if interested in receiving further information about participating in the study as a peer supporter or as a group member. General Practitioners (GPs), community health centres, pharmacies, diabetes clinics, podiatrists, optometrists and any active DA–Vic ComNet groups or other community based groups within the selected locations are also targeted with posters and brochures to increase study exposure.

Self-identified peer supporters and participants with an interest in the project are sent comprehensive explanatory statements, outlining the aims of the study and the randomisation process. Participants are asked to return a signed informed consent indicating their agreement to participate whilst acknowledging their ability to withdraw if desired.

### Diabetes self-management education

All enrolled participants (including identified peer supporters) are invited to attend one day of diabetes self-management education (DSME), held in ten locations around the state of Victoria. Education is delivered by a credentialed diabetes educator and accredited practising dietician from DA–Vic. The seven-hour program covers basic disease information, good self-management practice including diet and physical activity, disease complications and medications. A project specific education manual is given to each attending participant. Participants who cannot attend a full day program are sent the education manual and a copy of a DVD, filmed during one of the program education sessions and supplemented with visual material to reiterate DSME messages.

### Allocation

Upon completion of recruitment (participant and peer supporter), DSME and baseline measurement, groups are randomized in each health region (comprising 2 or 4 groups) so that each health region contained an equal number of intervention and usual care groups. Allocation to intervention or usual care was governed by a random number generation process using Stata statistical software, Release 11.

### Usual care

Participants and supporters from locations allocated to the control arm continue to receive their usual care and receive a copy of all anthropometric and biomedical measurements collected at baseline, 6 & 12 months with a recommendation to share these results with their clinical team.

### Intervention condition

**Figure 3:**
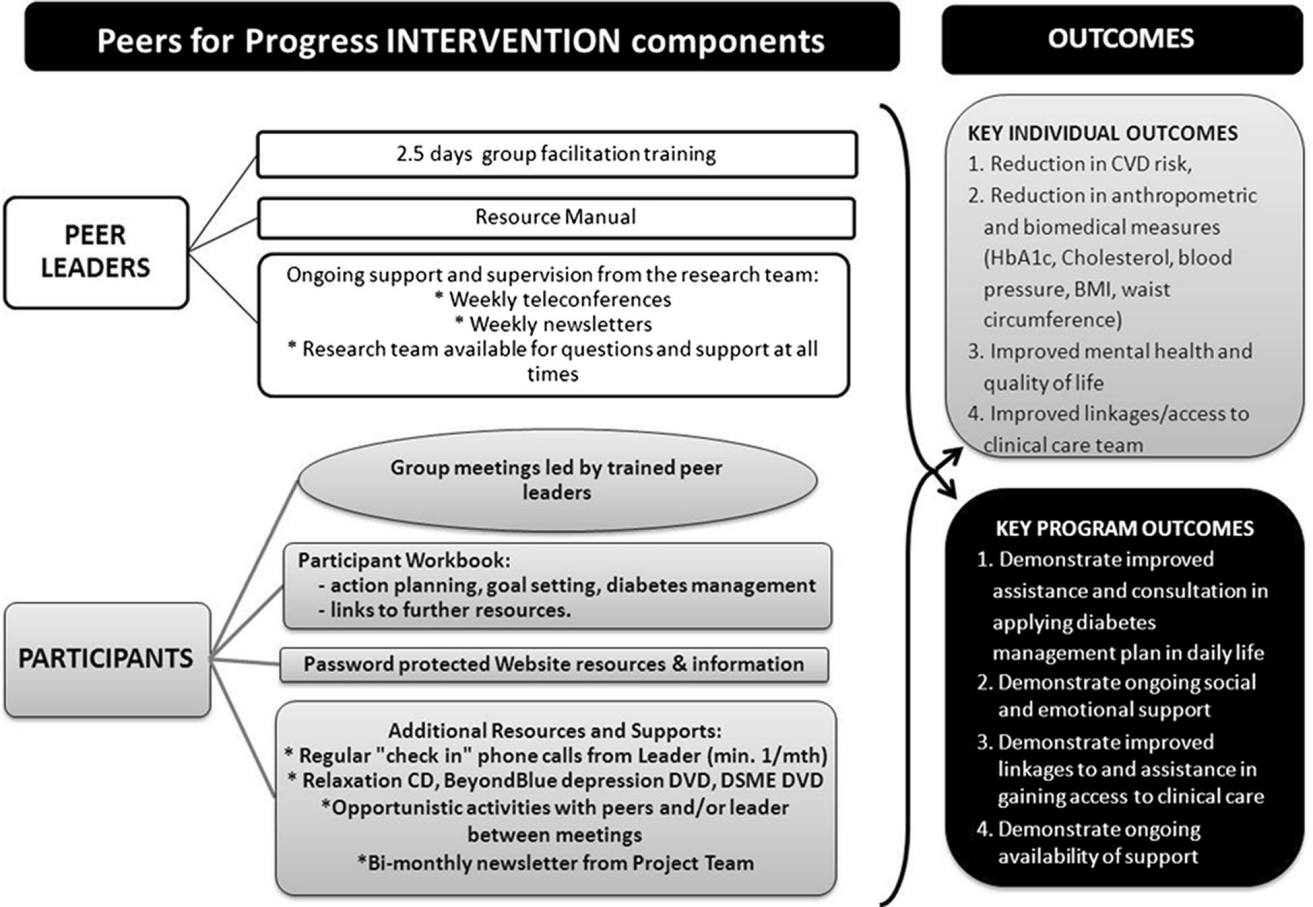
**Australasian Peers for Progress Diabetes Project Intervention components and outcomes**.

Figure 3 summarizes the different components of the peer support intervention program. This multi faceted intervention comprises four interconnected components to deliver support to the participants.

Monthly local community based meetings, facilitated by a trained peer supporter.

Regular contact between supporters and participants, in person or by phone, to offer support and assistance.

A manual and resource book, developed specifically for this program.

Access to a password protected website containing further information, resources and education vignettes.

Participants are encouraged and reminded by the supporter to attend all of the scheduled monthly group sessions during which the group members discuss and share challenges of diabetes management and support each other to set and achieve goals. Apart from meetings one and twelve, the content of the group meeting is flexible and is determined by the particular concerns and characteristics of individual groups. Suggested topics for discussion include risk assessment, goal setting, linkage to clinical care, diet, exercise, feet care, optical and mental health. The group members, according to their needs and interests, arrange visits to the group meetings by local health care professionals.

Providing support to each other through regular supporter-participant or participant-participant contact, between monthly sessions is promoted in order to maintain motivation and encourage self-improvement and confidence in diabetes self management. Participants and supporters are encouraged to meet socially, for shared activities (walks, exercise classes) and to motivate and encourage each other beyond the time spent together at the monthly meetings.

Participants receive a manual/workbook, to use during and outside the monthly meetings, which provides educational information including health recommendations and guidelines for diabetes management, helpful tips and advice for managing diabetes, information about available clinical care and tips on how to facilitate access to and use clinical care effectively. This manual also has space for recording goals, challenges, clinical measures and notes from discussion arising during the meetings.

Participants have access to a password protected website which offers additional resources to support group meeting discussions and activities. It provides links to other health and diabetes-related websites so that participants can easily navigate to relevant pages if they are looking for specific information regarding their diabetes management. It also contains recorded vignettes of the education session delivered to all participants prior to randomisation.

A bi-monthly newsletter “Support Share Learn Live” containing project news, support group profiles and photos, healthy recipes, and information to supplement meeting topics is posted to all intervention participants.

Supporters are asked to maintain and record; using a monthly contact record sheet, email or phone contact with group members throughout the month at which time they may inform participants about upcoming meetings or group activities as well as provide additional support. The monthly contact record also requires supporters to provide details of discussions and topics covered at each meeting.

### Peer supporter training & support

Suitable peer supporters from locations randomized to the intervention arm are trained over two and a half days by a credentialed diabetes nurse educator experienced in group facilitation training. Peer supporter training aims to equip supporters with communication and group facilitation skills to help them support their group members to tell their stories, set goals, problem solve, increase awareness and linkages with the available health system, optimize self-management behaviours, including glucose monitoring, dietary changes and physical activity, as well as provide emotional support. The work with the research team with respect to data collection including reports of group meetings and individual contacts is reviewed and explained during the training. Additionally, peer supporters are invited to assist with scheduling meetings and to locate potential venues for group meetings within their communities.

Peer supporters are supported during the intervention period through weekly teleconferences with the project and specialist staff including diabetes educators, dieticians and psychologists. Furthermore, peer supporters receive a weekly informational e- newsletter about aspects of diabetes care and self-management, additional information in response to questions from group members and information they might share with their group members as well as reminders about joining the teleconference the following week and minutes from the preceding teleconference (Figure 3). Peer supporters are asked to attend at least one teleconference each month. These calls, which are recorded, emulate a supportive peer group meeting in that discussion and conversation with the project staff will provide organisational and informational support and the supporters provide experiential, emotional and social support to each other. During these teleconferences, supporters review their group meetings, discuss important events, share stories, ask questions on behalf of their group members and receive support from their peers.

### Data collection and outcome measures

Baseline data collection is completed prior to randomization and is accomplished by a self-administered survey, anthropometric measurements collected by the study team or obtained from the participant’s clinical care provider as well as blood and urine analysis, specifically for the study or from a recent test requested by the clinical care provider. Table 1 shows the measurement tools used for data collection, the method of collection and time points (baseline, six or 12 months) at which data is collected. Anthropometric measures are collected by project staff specifically trained in accordance with standard clinical measurement methods [21]. Briefly, the left arm circumference is measured to determine the appropriate cuff size for blood pressure (BP) measurement. BP and pulse is measured and recorded three times using the Omron HEM-907 IntelliSense professional digital blood pressure monitor with at least one minute between readings. Height is measured using a portable stadiometer height rod to the nearest 0.5 cm whilst the participant is standing without shoes. Weight is measured to the nearest 0.1kg using a Salter portable weighing scale, model 913. Waist circumference is measured halfway between the lowest rib and the top of the hipbone using the Novel figure finder tape measure. Duplicate waist circumference measurement is undertaken for approximately 10% of the participants for quality assurance. Blood pressure machines and weight scales are calibrated at least weekly. Pathology laboratories providing blood and urine analysis are accredited by the National Association of Testing Authorities (http://www.nata.asn.au/) and all methods for determining HbA1c% are NGSP approved (http://www.ngsp.org/). 

**Table 1:** Measurement domains, survey tools and collection method used at each data collection time point

**Variable**	***Component***	**Measurement tools/Questions**	**Base line**	**6 mth**	**12 mth**	**Type of administration**
Demographic Measures		Age at diagnosis	✓	✓		
		Health Insurance	✓		✓	Postal survey
		Age, Sex, Country of birth, Living Arrangement (Marital status), Education, Income, Employment status,	✓		✓	
		Ethnicity, Health Care Access,	✓		✓	
Clinical Information	*Current Clinical Measures*	On insulin, since when, #/day, Most recent HbA1c,	✓	✓	✓	
		Other medical treatments, past medical history	✓		✓	Postal survey
		List of prescribed medications, last dilated eye				
		exam/retinal photo, foot exam, Past medical history,	✓	✓	✓	
	*Health before, at and since diagnosis*	Pre-diagnosis; at diagnosis and post-diagnosis conditions	✓		✓	Postal survey
	*Anthropometric*	Waist circumference *(Novel Figure Finder Tape Measure)*	✓	✓	✓	Collected by project team /or from clinical care provider (CCP)
		Blood Pressure *(Omron HEM-907 IntelliSense*	✓	✓	✓	
		*professional digital blood pressure monitor)*				
		Weight *(Salter portable weighing scale, model 913)*	✓	✓	✓	
		Height *(Portable Stadiometer Height Rod)*	✓			
	*Pathology*	HbA1c,Total Cholesterol(TC), TC/HDL ratio	✓	✓	✓	Pathology for study/ CCP
		Urine microalbumin, Fasting blood glucose, Trig, HDL, LDL,	✓		✓	
Behavioural	*Physical Activity*	Single physical activity question (# minutes/week)	✓	✓	✓	Postal survey
	*Nutrition*	Cancer Council Food Frequency Questionnaire [22]	✓		✓	FFQ
	*Smoking*	Current/ever smoked/ FFQ /SDSCA	✓	✓	✓	Postal survey
	*Alcohol*	Alcohol days/ quantity/ FFQ	✓	✓	✓	Postal survey
	*Self-care activities*	(SDSCA) Selfcare activities scale with Toobert, Fisher & Glasgow modifications [23,24]	✓	✓	✓	Postal survey
	*Medication*	Morisky 8-item [25,26]	✓	✓	✓	Postal survey
QOL	*Assessment of quality of life*	AQoL-8D [27] and EQ-5D™[28,29]	✓	✓	✓	Postal survey
	*Diabetes Distress*	Diabetes Distress Screening Instrument: DDS4 [30,31]	✓	✓	✓	Postal survey
	*Depression*	PHQ-9 [32]	✓	✓	✓	Postal survey
Mediators and Moderators	*Health literacy*	[33]	✓	✓	✓	Postal survey
	*Non-directive vs directive support*	[12]		✓	✓	
	*Availability & satisfaction with diabetes-support from family, friends & health care team*	[34]	✓	✓	✓	Postal survey
	*Sources of Support*	Who are you supported by?	✓	✓	✓	Postal survey
	*Diabetes Knowledge*	Michigan Diabetes Research and Training Centre with modification to Australian context [35]	✓	✓		Postal survey
	*Self-Efficacy*	Diabetes Self-Efficacy Scale [36]	✓	✓	✓	Postal survey
Use of diabetes services	Health-care utilisation	GP details, Travel time, waiting time	✓	✓		Postal survey
		Insurance, NDSS membership, DA-Vic membership, GP/ specialists/ health professionals visits over past 6 mths, ECG, vaccinations, hospital stays, etc. based on RACGP guidelines for T2DM management				
		Most recent HbA1c (self-report)				
		Cost of services				
Clinical services	*Health care utilisation*	GPAQ ver 2.1 [37]	✓		✓	Postal survey
		Clinical care agreements (annual cycle of care, General				
		Practice Management Plan, Team Care Arrangement)	✓	✓	✓	Postal survey
Cost Effective Analysis		# visits past 6 mths to diabetes clinician	✓	✓	✓	Postal survey
		# visits past 6 mths to other clinicians	✓	✓	✓	
		# visits past 6 mths for emergency/acute care	✓	✓	✓	
		# overnight stays past 6 mths in hospital (related to your diabetes)	✓	✓	✓	
Peer Training/ Quality Improve.	*Training effectiveness*	Usefulness of training		✓	✓	Postal survey
		Extent of content covered				
Process Evaluation		17 item assessment with 3 additional questions regarding most helpful, least helpful and suggestions to improve		✓	✓	Postal survey
		+ 2 questions asking availability of participant to other group members [38-40]				

The primary outcome measure is cardiovascular disease (CVD) risk using the UKPDS risk calculation [16]. People with diabetes are already at higher risk of CVD than the general population without diabetes. We consider that a 10% reduction in risk will be clinically significant, if we assume that the average risk for our participants is likely to be 20 – 25% in line with that in a sample with type 2 diabetes from Australian general practice [41]. Age at diagnosis, sex, ethnic group, smoking status, HbA1c, systolic blood pressure and total cholesterol/HDL ratio will be used in the UKPDS equation to determine CVD risk at baseline and 12 months [16]. Changes in the CVD risk over time as well as changes in waist circumference, body mass index (BMI), weight, and lipid profile will be used to assess diabetes self-management post intervention. Quality of life (AQoL [27] & EQ-5D[28,29]), depression (PHQ9, [32]) diabetes distress screening [30,31], self-care activities [23,24], medication adherence [25,26], food, smoking and alcohol intake [22], current support systems [34], known co-morbidities associated with diabetes, utilization of and satisfaction with available health services [37], diabetes knowledge using the Michigan Diabetes Research and Training Center Brief Diabetes Knowledge Test with modification to Australian context [35], health literacy [33] and confidence of diabetes management [36] are measured at baseline, six and 12 months.

### Program evaluation

Glasgow’s RE-AIM framework is being used to evaluate the program [42]. We are evaluating individual, group, and system level measures to assess the success of the program in achieving the key functions of peer support as specified by the global Peers for Progress Program Center [11] i.e. Assisting daily management, improving linkage to clinical care, providing social and emotional support and provide ongoing support. A process evaluation will determine the implementation fidelity of the trial and will assist in future refinements of the Aust PfP-DP.

#### Group-level measurements

In addition to individual-level measurements as outlined in Table 1, we will also measure group- and facilitator-level outcomes, including group effectiveness, satisfaction of group members and peer supporters and team dynamics, using a range of questions assembled in collaboration with the global Peers for Progress group [11] and based on items generated by Fisher in the Diabetes Initiative [43] and items from resources and supports for self-management (RSSM) and Patient Assessment of Chronic Illness Care (PACIC) [38-40]. Participants and supporters in the intervention arm are asked to complete an evaluation of the resources, health care providers who visited the groups and group meetings and perceptions of support received by supporters or other group members.

#### System-level evaluation

Feasibility of further dissemination and implementation of the Aust PfP-DP will be evaluated according to the five key dimensions of the RE-AIM framework which includes systematic measurement of internal validity (i.e. the ability of the intervention to produce a sustained change in patient health behaviours, including **E**ffectiveness and **M**aintenance) and external validity (i.e. the potential for the intervention to be generalized to other settings including **R**each, **A**doption, and **I**mplementation) [42]. Outcomes such as increased utilisation of DA-Vic resources, memberships and expansion of groups as a result of the intervention will also be assessed.

### Economic evaluation

The economic evaluation of the Aust PfP-DP will evaluate whether the program delivers ‘value-for-money’. This will be undertaken through a ‘trial-based evaluation’ (costs and outcomes exactly as per the trial), as well as a ‘modelled economic evaluation’, which will extend the target population, time horizon and decision context.

The cost data will also be combined with the change in the quality of life score for participants using the AQoL-8D [27] in both the trial-based and modelled evaluations. This will be helpful to consider value-for-money against a reference threshold (e.g. $50,000 per QALY).

The modelled evaluation will use the UKDPS outcomes model [44] to translate the changes in clinical outcomes observed over the relatively short time horizon of the study into potential lifetime reductions in cardiovascular and other diabetes related risks. The cost implications of the changes in health status will be estimated by drawing on Australian disease specific health expenditure data.

### Data analysis

Analysis will be performed based on based on an intention-to-treat i.e. without regard to the compliance of individuals within their allocated study arm. Analysis of covariance (i.e. linear regression models for 12-month measurements with baseline scores and intervention arm as co-variates) will estimate the changes from baseline to 12 months that can be attributed to the peer support intervention. Analyses will be done firstly on all individuals who complete follow-up and secondly on all individuals who were randomized using multiple imputation principles to deal with the missing data in individuals who do not complete follow-up. All analyses will be carried out with Stata software. Regression analysis will be used to determine the extent to which a variety of individual-, group- and system-level characteristics are associated with effective implementation and outcomes.

## Discussion

This article provides a comprehensive description of the methods being used to implement and evaluate a complex community-based randomized controlled trial of group peer support for people with type 2 diabetes in Victoria, Australia. The successful implementation of this trial will provide evidence about the key functions of peer support identified by the global Peers for Progress program.

## Competing interests

The authors declare they have no competing interests.

## Authors' contributions

MR contributed to study design, managed the project, directed data collection and prepared the first draft of the manuscript, PfP Principal Investigators (BO, JD, PR, PA, RC, GJ, RW, MdeC, AZ and VH) conceived of the proposal, obtained funding and contributed to the study design, preparation of the manuscript and approved the final draft , CR participated in funding application, study design, recruitment, data collection and earlier drafts of the manuscript, SC contributed economic analysis design and data collection methods. All authors read and approved the final draft.

## Authors’ information

Principal Investigator: Professor Brian Oldenburg (Monash University). Investigators: Adjunct Professor Pilvikki Absetz (National Institute of Health and Welfare (THL) Finland), Professor Rob Carter (Deakin University), Associate Professor Max de Courten (University of Copenhagen), Professor James Dunbar (GGT UDRH, Flinders University & Deakin University), Virginia Hagger (Diabetes Australia - Victoria), Adjunct Professor Greg Johnson (Diabetes Australia – Victoria & Deakin University), Professor Prasuna Reddy (Centre for Rural and Remote Mental Health (CRRMH), University of Newcastle) Associate Professor Rory Wolfe (Monash University) & Professor Anuar Zaini (Monash University, Malaysia).

## Pre-publication history

The pre-publication history for this paper can be accessed here:

http://www.biomedcentral.com/1471-2458/12/843/prepub
